# Supplementary material for: Phototoxic effects of nonlinear optical microscopy on cell cycle, oxidative states, and gene expression
Source: Sci Rep. 2022 Nov 5;12:18796. doi: 10.1038/s41598-022-23054-7 (PMC9637160; doi:10.1038/s41598-022-23054-7)
Supplement: Supplementary file 1 — Supplementary Information. [file 41598_2022_23054_MOESM1_ESM.pdf]

**Supplementary information**

**Phototoxic effects of nonlinear optical microscopy on cell cycle, oxidative states, and gene expression**

Xinyi Zhang[1,*], Gabriel Dorlhiac[2], Markita P. Landry[2,3,4], Aaron Streets[1,2,4]
1. University of California, Berkeley, Department of Bioengineering, Berkeley, CA
2. University of California, Berkeley, Biophysics Graduate Group, Berkeley, CA
3. University of California, Berkeley, Department of Chemical and Biomolecular Engineering, Berkeley, CA
4. Chan-Zuckerberg Biohub, San Francisco, CA
* Current address: Massachusetts Institute of Technology, Department of Electrical Engineering and Computer Science, Cambridge, MA

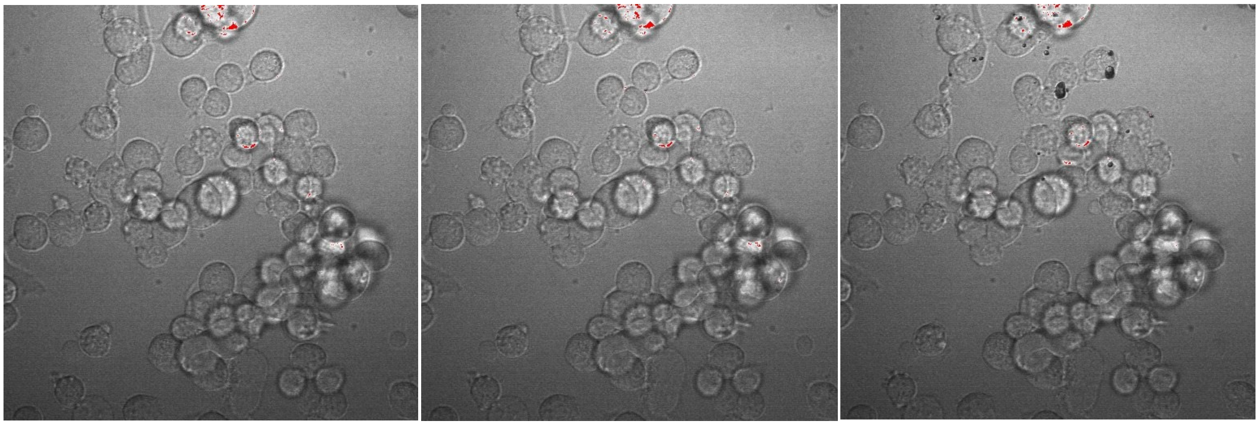

**Figure S1**. 2nd, 14th, and 51st frames of N2A cells exposed to near-burning condition (I3-E3). I3: average intensity = 397 mW/µm$^2$; E3: fluency = 79.4 J/cm$^2$.

(a)

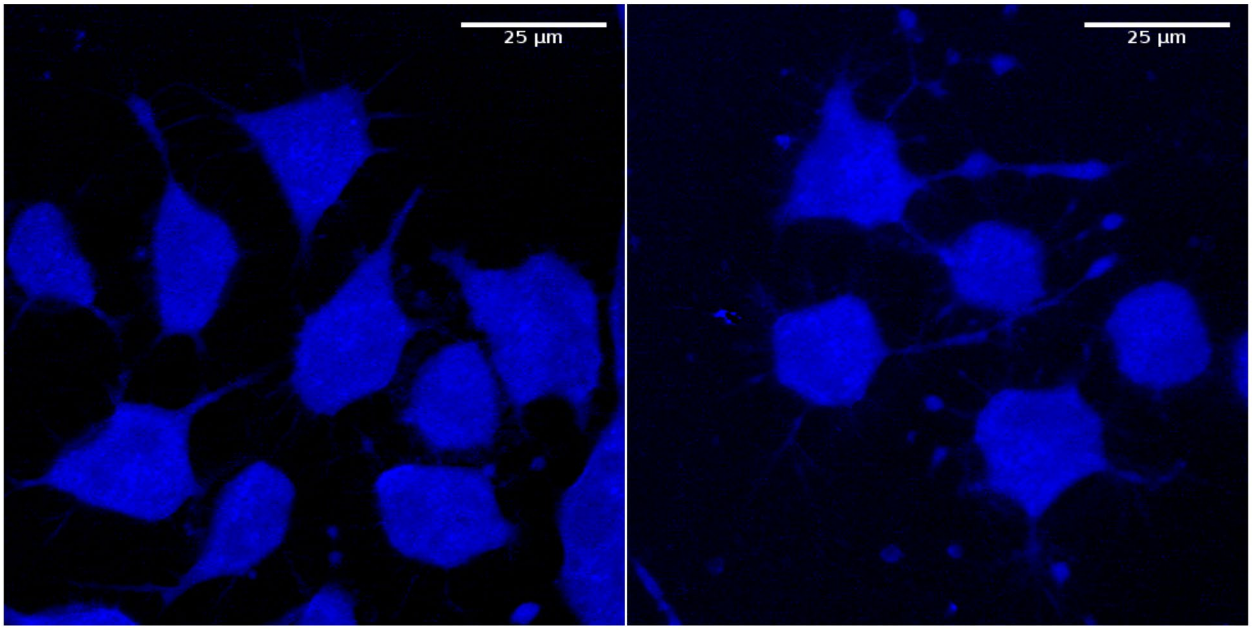


**Figure (S2):** Representative SRS Images of N2A Cells at 2950 cm-1
(a) SRS images acquired of N2A cells on slide-mounted coverslips using the 60x, 1.2 NA, water immersion objective. Images were acquired at 2950 cm-1, the CH3 stretching band, which is dominated by signal contributions from cellular protein. A Raman shift of 2950 cm-1 corresponds to a pump wavelength of 796 nm and a Stokes wavelength of 1040 nm. The power of both the pump and Stokes lasers was set to 10 mW. A pixel dwell time of 10 µs was used for acquisition.

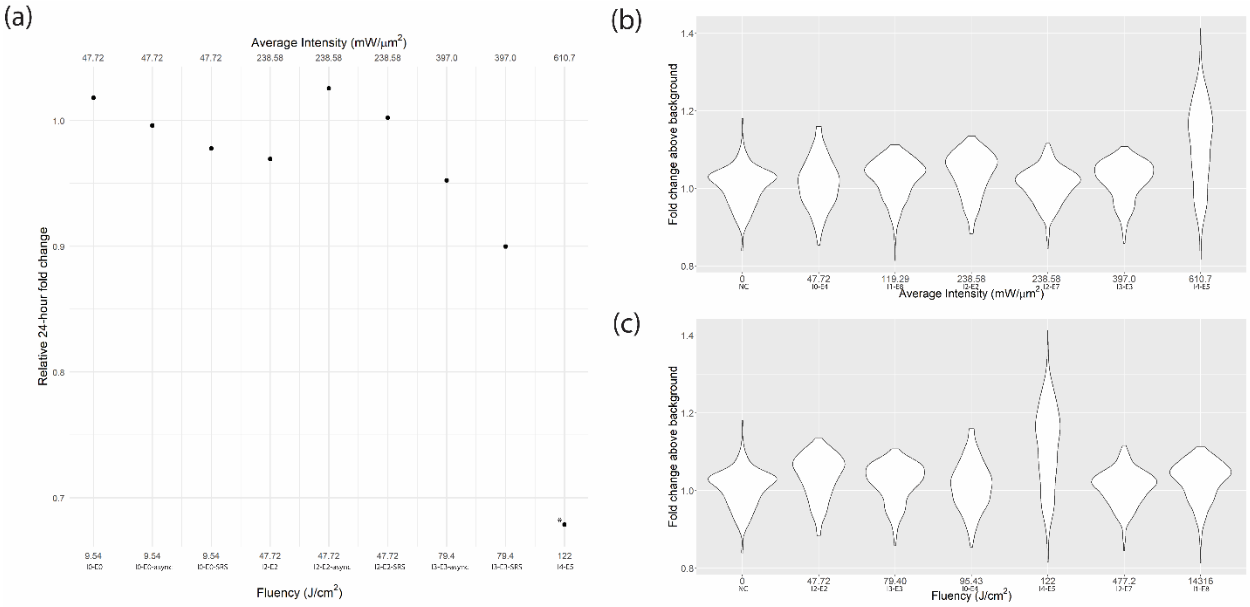


**Figure S3**. (a) Proliferation of each imaging condition after 24 hours of post-exposure incubation divided by the proliferation of the negative control (NC) samples. *: p-value < 0.05. (b)-(c): Distribution of mean intensities of all cells in samples with laser exposure and the negative control group after flatfield correction. These are the same data as in Figure 4b and are ordered by either increasing average intensity (b) or increasing fluency (c).

**Supplementary Table 1.** Summary of damage thresholds in this study and reported in the literature. The damage threshold is the minimum average intensity or the minimum fluency under which the specific damage effect can be observed.

| Study | Cell type | Intensity Threshold (mW/µm^2) | Fluency threshold (J/cm^2) | Peak Intensity threshold (W/µm^2) | damage effect/observation |
|---|---|---|---|---|---|
| This study | N2A | 611 | 122 | 63615 | ROS |
| | N2A | 611 | 122 | 63615 | Proliferation |
| | N2A | >397 | >95 | >41354 | DEG |
| REF 11 | CHO | 75 | 4485 | 3893 | propidium iodide |
| | CHO | 205 | 12288 | 1164 | propidium iodide |
| | CHO | 46 | 2765 | 2400 | 50% cloning efficiency |
| | CHO | 75 | 4485 | 425 | 50% cloning efficiency |
| REF 12 | myelin sheath | 20 | 457 | 1023 | bright plasma emission |
| | KB cells | 189 | 4329 | 9700 | membrane blebbing |
| REF 13 | Ptk2 | 50 | 2981 | 5175 | ROS |
| | Ptk2 | 142 | 8516 | 14784 | propidium iodide |
